# Regulation of Cisplatin Cytotoxicity by Cu Influx Transporters

**DOI:** 10.1155/2010/317581

**Published:** 2011-01-09

**Authors:** Paolo Abada, Stephen B. Howell

**Affiliations:** Moores Cancer Center and Department of Medicine, University of California, San Diego, La Jolla, CA 92093, USA

## Abstract

Platinum drugs are an important class of cancer chemotherapeutics. However, the use of these drugs is limited by the development of resistance during treatment with decreased accumulation being a common mechanism. Both Cu transporters CTR1 and CTR2 influence the uptake and cytotoxicity of cisplatin. Although it is structurally similar to CTR1, CTR2 functions in a manner opposite to that of CTR1 with respect to Pt drug uptake. Whereas knockout of CTR1 reduces Pt drug uptake, knockdown of CTR2 enhances cisplatin uptake and cytotoxicity. CTR2 is subject to transcriptional and posttranscriptional regulation by both Cu and cisplatin; this regulation is partly dependent on the Cu chaperone ATOX1. Insight into the mechanisms by which CTR1 and CTR2 regulate sensitivity to the Pt-containing drugs has served as the basis for novel pharmacologic strategies for improving their efficacy.

## 1. Introduction

Platinum- (Pt-) based chemotherapeutic agents have been utilized in the treatment malignancies since their first approval in the 1970s. Cisplatin, carboplatin, and oxaliplatin are the three most commonly used Pt drugs in the USA and are an essential part of the standard of care for lung, ovarian, colorectal, testicular, bladder, and head and neck cancer as well as other malignancies. Unfortunately the use of these compounds is limited by both acute and cumulative toxicities as well as the development of resistance. The Pt-containing drugs are believed to function *in vivo *by forming DNA adducts that lead to apoptosis. The mechanisms that account for primary or acquired resistance to the Pt-containing drugs continue to be the subject of intense study given the clinical utility of these compounds. Resistance appears to be multifactorial, and differences in DNA repair, detoxification, and drug accumulation have all been implicated [1, 2]. Decreased accumulation is one of the most commonly observed features observed in resistant tumor cells both *in vitro* and *in vivo,* and there are now multiple lines of evidence indicating that proteins involved in Cu homeostasis are responsible for the import, intracellular distribution, and export of the various Pt agents [1]. This short paper will focus on recent developments in understanding how the cellular pharmacology of the Pt-containing drugs is influenced by proteins belonging to the Cu homeostasis system with an emphasis on the Cu transporter (CTR) family and Cu transporter 2 (CTR2) in particular.

## 2. Overview of Cu Homeostasis

Cu is essential for the functioning of multiple cellular enzymes including superoxide dismutase, cytochrome *c* oxidase, lysyl oxidase, and dopamine *β*-hydrolase [3, 4]. These enzymes are required for crucial cellular processes such as electron transport and detoxification of reactive oxygen species. The reactions mediated by these enzymes involve the interconversion of Cu between two different oxidation states, Cu(I) and Cu(II). However, the ability of Cu to undergo these redox reactions under physiologic conditions makes it extremely toxic if left free within the cell. For this reason, cells have developed a complex system of Cu transporters and chaperones that bind Cu and protect it from oxidation such that there is essentially no free intracellular Cu (less than 10^−18^ M). The components of the Cu transport systems are highly conserved in biological organisms from bacteria to humans, a feature that further highlights the importance of this system.

A common characteristic of Cu transport proteins and chaperones is the presence of methionine, histidine, or cysteine-rich motifs capable of chelating Cu. Cu and other metal ions bind to these motifs through electrostatic interactions which allow the transfer of a metal ligand either from one domain to another of a single protein, or from one protein to another, via transchelation reactions. Influx transporters are found in the plasma membrane that deliver Cu to cytoplasmic Cu chaperones that, in turn, transfer Cu to either other Cu chaperones, Cu requiring enzymes, or other transporters that concentrate it into the trans-Golgi network and the secretory pathway. 

**Figure 1:**
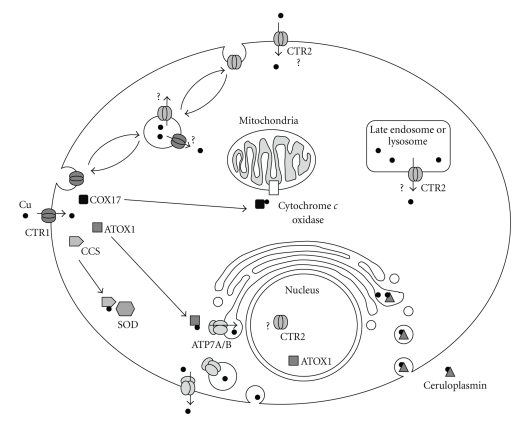
Schematic diagram of Cu homeostasis.

Extracellular Cu(II) bound to ceruloplasmin is believed to be converted to Cu(I) by cell surface reductases homologous to yeast FRE1 and FRE2. Cu then enters the cell as Cu(I) in a non-energy-dependent process through a pore created by a homotrimer of the high affinity transporter CTR1 [5–7]. Mammalian cells also express a second Cu transporter, CTR2, that has lower affinity for Cu. It may also influx Cu across the plasma membrane [8], although studies in yeast suggest its primary role is to export Cu from the vacuole which serves as a Cu storage site [9, 10]. After entry into the cell, Cu is passed to the chaperones ATOX1, COX17, and CCS each of which then transfers Cu to a specific enzyme complex (Figure 1). COX17 delivers Cu via additional chaperones to cytochrome c oxidase whereas CCS delivers it to superoxide dismutase. ATOX1 transfers Cu to the P-type ATPases ATP7A and ATP7B which concentrate Cu into the trans-Golgi for incorporation into ceruloplasmin and other Cu containing proteins present in the secretory pathway. ATOX1 has recently also been shown to function as a Cu-sensitive transcription factor [11]. In the setting of excess levels of Cu, delivery of Cu to ATP7A and ATP7B triggers their trafficking to the plasma membrane or vesicular structures near the cell surface from which Cu is eventually exported [12–14]. Cu can also form complexes with glutathione from which it is delivered to metallothionein II. Recent work by Banci et al. [15] showed that the individual affinities of the various Cu containing chaperones and enzymes are such that they provide a gradient that can drive Cu from the lower affinity chaperones and storage sites to the higher affinity enzymes.

## 3. Characteristics of CTR1

**Figure 2:**
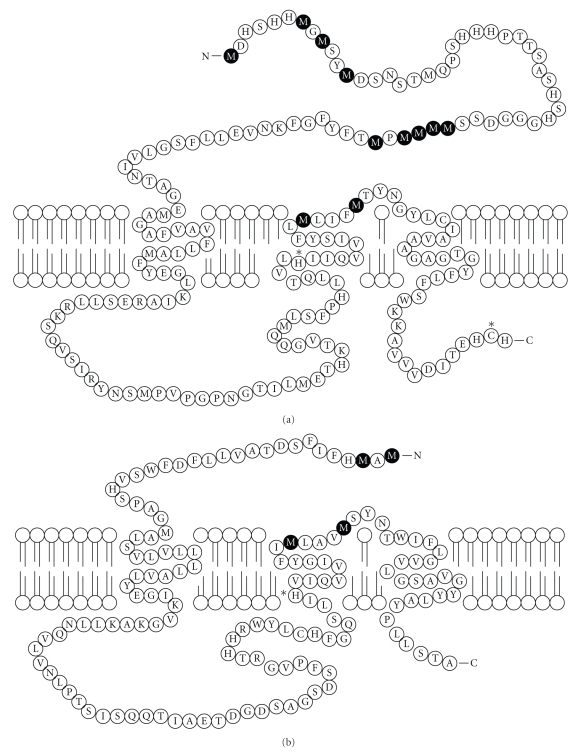
Schematic diagram of hCTR1 and hCTR2 showing membrane topology and sequence. Important methionine residues within the metal binding sequences and transmembrane regions are shown as darkened residues. The asterisk denotes other amino acids (H139 and C189) important for Cu transport.

CTR1 was first identified in yeast as necessary for high affinity Cu import in the mid-1990s [5]. It is a highly conserved protein as demonstrated by the fact that both human and mouse CTR1 can rescue the growth defect produced by knocking out CTR1 in *S. cerevisiae* [6, 16]. Human CTR1 contains 190 amino acids organized into three transmembrane domains, an N-terminal extracellular domain rich in methionines and histidines, a large intracellular loop, and a short intracellular C-terminal tail (Figure 2(a)). Conserved methionine-containing motifs and individual methionines, histidines, and cysteines essential to Cu transporter function are found within the extracellular domain, within the second and third transmembrane domains, as well as in the C-terminal tail. CTR1 forms a homotrimer in the membrane, and structural studies suggest that it assembles into an inverted cone-shaped pore through which Cu(I) is transmitted from one side of the membrane to the other [17]. Recent computational studies provide further support for the importance of several of these conserved residues in transporter function, particularly within the second transmembrane domain [18].

## 4. Regulation of Pt Drug Cytotoxicity by CTR1

The Cu influx and efflux transport proteins were first linked to the import of the Pt-containing drugs when it was noted that cells selected for resistance to cisplatin were cross-resistant to various other metals including Cu [19–23]. Cells selected for resistance to Cu were found to be cross-resistant to cisplatin and vice versa. Currently there is evidence that changes in the expression of influx transporters CTR1 and CTR2, the chaperone ATOX1, and the efflux transporters ATP7A and ATP7B alter sensitivity to the cytotoxic effect of the Pt-containing drugs [21, 24–26]. All these proteins contain metal binding motifs which, in addition to binding Cu, are likely to interact with the Pt drugs as evidenced by the results of crystallographic and mass spectrometry experiments [27–29]. 

The evidence that CTR1 functions as an importer for the Pt-containing drugs has been reviewed recently [30], and only the key points will be summarized here. CTR1-deficient yeast were noted to be resistant to both Cu and cisplatin due to impaired uptake [24, 31], and subsequent studies in mouse embryo fibroblasts in which both alleles of CTR1 were knocked out documented that the influx of all three clinically used Pt-containing drugs was impaired. The largest effect of CTR1 appears to be on initial influx, although a recent study also identified enhanced efflux in CTR1^−/−^ cells [25]. Knockout of CTR1 results in nearly complete resistance to the therapeutic effect of cisplatin when mouse embryo fibroblasts are grown as xenografts in nu/nu mice [26]. More recent work has focused on the mechanism by which CTR1 imports the Pt drugs. Current evidence suggests that the Cu atom is passed through the pore formed by the CTR1 trimer. Key to this function is the conserved ^40^MMMMPM^45^ motif located proximal to the start of the first transmembrane domain, and methionines M150 and M154 in the second transmembrane domain, both of which are required for optimal Cu uptake. When assembled as a trimer, these residues appear to form rings of methionines which line the channel and mediate the passage of Cu through a series of transchelation reactions. The conserved cysteine in the C terminus (C189) may also play a role in Cu transport as there is evidence that it binds Cu and may serve as part of a gating mechanism for the channel [32]. The selectivity of CTR1 for Cu(I) rather than Cu(II) may result from differences in their coordination chemistry and the strength of the bond formed with the methionines. The stronger bond formed by Cu(II) may limit the ability to undergo the transchelation reactions proposed to be essential for uptake. In support of this, it has been recently reported that silver, whose affinity for the sulfur of methionine is similar to that of Cu(I), is transported by CTR1 [33]. 

The details of how the Pt drugs pass through the CTR1 pore are still lacking. Although the Pt-containing drugs are significantly larger in size than Cu, it is possible that trimeric CTR1 is quite expandable and may be able to accommodate these molecules as they pass through. Recent computer modeling studies of the transmembrane helices predict that these structures are quite dynamic, with some critical portions, such as the transmembrane methionines at 150 and 154 in the second transmembrane domain, serving as hinges for the overall structure [18]. The relative stability of these “hinge” methionines make them a potential bottle neck in the passage of a Cu atom through the pore and may in fact be essential to how the cell controls Cu influx. The affinity of cisplatin for the sulfur of methionine is similar to that of Cu(I), and it has been theorized that it may pass through the trimer via a series of transchelation reactions in a manner similar to Cu [30]. However, two recent studies noted that cisplatin reacts with methionine-containing sequences quite slowly relative to its rate of cellular uptake and that both ammine ligands are lost when cisplatin reacts with either ATOX1 or a peptide containing a methionine motif similar to that found in the N-terminal domain of CTR1 [27, 29]. Since the ammine ligands are present in the DNA adducts formed by cisplatin and reacquisition of ammine ligands intracellularly once lost is unlikely, if cisplatin is passing through the pore it must do so in a manner that retains the ammines. Other recent work indicates that there are substantial differences in the way in which CTR1 transports Cu and cisplatin. For example, conversion of the methionines at 150 and 154 to isoleucines, while impeding Cu transport, actually increases the uptake of cisplatin [34]. The CTR1 conformation that best accommodates Cu may not be the best for cisplatin. H139 is known to affect the ability to transport Cu. Mutation of this residue impairs Cu uptake, and the computational model suggests that the interaction of this residue with G84 stabilizes an active Cu transporting conformation. With regards to cisplatin, mutation of H139 resulted in an increase in cisplatin transport suggesting that this lifts restrictions that allow the pore to adopt conformations more amenable to cisplatin transport but which are detrimental to Cu flux.

The role of the most N-terminal histidine and methionine motifs in CTR1 with respect to the transport of either Cu or the Pt-containing drugs has not been well defined. When Cu is abundant, these motifs are not required for Cu uptake although they enhance transport when Cu is scarce [35–38]. The Pt-containing drugs are capable of interacting with metal binding motifs such as those found in the N-terminal region of CTR1, and deletion of the ^40^MMMMPM^45^ motif, or of the entire first 45 amino acids, impairs cisplatin uptake [28, 39, 40]. Quite possibly these N-terminal motifs serve to funnel Cu and cisplatin towards the transmembrane pore. 

## 5. Characteristics of CTR2

yCTR2 was identified on the basis of its ability to rescue the growth of *S. cerevisiae* that were Cu deficient due to defective yCTR1 and yCTR3, the normal high affinity membrane transporters in this organism [41]. Initially it was thought that yCTR2 functioned as a low affinity Cu influx transporter at the cell surface. Work by Portnoy et al. [10] and Rees et al. [9] showed that in yeast yCTR2 was primarily localized to vacuolar membranes. Deletion of the gene coding for yCTR2 increased the vacuolar Cu level suggesting that yCTR2 functions to release vacuolar stores of Cu when cytoplasmic Cu is low. Export of Cu from these intracellular stores through yCTR2 was found to be dependent on the vacuolar Cu reductase Fre6 that is likely needed to convert vacuolar Cu(II) to Cu(I) permitting it to be transported by yCTR2 [42]. Fre1 and 2 at the yeast plasma membrane serve a similar role in reducing Cu(II) to Cu(I) prior to transport by yCTR1 [42]. However, a variant of yCTR2 was found that mislocalized to the cell surface. This variant, identified as yCTR2-1, contained two mutations, a tryptophan to arginine mutation at position 7 and a C terminal truncation of the last 16 amino acids of yCTR2. When thus localized to the plasma membrane yCTR2-1 was capable of importing extracellular Cu and of reversing the growth impairment of yeast deficient in both yCTR1 and yCTR3 [9, 42]. 

In mammalian cells the majority of CTR2 is localized to the late endosome and lysosome compartments with little present at the cell surface; there may be some colocalization with CTR1 at intracellular sites [43, 44]. This would suggest that, similar to yCTR2, mammalian CTR2 functions to release intracellular stores of Cu as these structures are similar in function to the yeast vacuole. However, there is also evidence in mammalian systems that CTR2 can function as a Cu influx transporter. Van Den Berghe et al. [43] found that overexpression of CTR2 in HEK 293 cells increased the expression of a reporter sensitive to intracellular Cu levels. Bertinato et al. [8] also found that forced expression of CTR2 mediated Cu import in the presence of high concentrations of extracellular Cu and that siRNA-mediated knockdown of CTR2 reduced Cu uptake. The affinity of CTR2 for Cu calculated from these studies (*K*_*m*_ ~6–11 *μ*M) was lower than that for CTR1 (*K*_*m*_ ~1.7 *μ*M) [8]. However, both of these studies utilized cells in which CTR2 was over-expressed which may have resulted in abnormalities of distribution with an unusual amount of CTR2 directed to the cell surface. In addition, these studies also used C-terminally tagged forms of CTR2, and this could have interfered with normal trafficking although no clear alteration in distribution was noted. It is of interest that the C-terminal end of CTR2 contains a conserved double leucine which mediates the endocytotic retrieval of many transmembrane proteins from the plasma membrane. It has already been shown in yeast that yCTR2-1 mutant which was missing the C-terminal 16 amino acids is mislocalized to the cell surface and can mediate extracellular Cu transport, and it is possible that similar phenomenon could have occurred in these studies. No information is currently available on the ability of CTR2 to export Cu from the endosomal/lysosomal compartment in mammalian cells. 

Given the paucity of studies on CTR2 to date, much of the structure-function relationships in CTR2 must be inferred from studies done on CTR1. CTR2 has significant structural homology to CTR1 although it only shares ~41% amino acid sequence homology. Similar to CTR1, CTR2 has an extracytoplasmic N-terminus, three transmembrane domains, a large intracellular loop, and a short intracellular C-terminus (Figure 2(b)). It also contains a conserved ^1^MXM^3^ motif within the N-terminus, approximately 23 residues from the first transmembrane domain, as well as conserved methionines in the second transmembrane domain in the form of ^111^MXXXM^115^. However, it is missing the more distal N-terminal metal binding domains and the cysteine that is present in the penultimate position in the C-terminal tail of CTR1. CTR2 forms oligomeric complexes, and given the structural homology to CTR1, it is presumed that CTR2 forms homotrimers which may function as a channel [43]. Mutation of the extracellular N-terminal methionines at residues 59 and 61, or the second transmembrane M148 and M152 of yCTR2, resulted in a protein that was unable to rescue the Cu deficiency of a ΔCTR1 ΔCTR3 ΔCTR2 yeast strain, and this was believed to be due to inability of the mutant yCTR2 to transport Cu [9]. The equivalent methionine residues are also known to be important in the Cu transport function of CTR1 and strongly suggest that CTR2 is capable of transporting Cu by a similar mechanism. Presumably the rings of methionines formed when the trimeric complex is assembled are involved in coordinating metal atoms as they pass through the CTR2 pore. Further, the ability of yCTR2 mutants to transcomplement each other also supports the supposition that CTR2 forms homo-oligomers, presumably a trimer similar to CTR1 [9]. 

As in yeast, when the methionines in the ^111^MXXXM^115^ motif in the second transmembrane domain of mammalian CTR2 were converted to ^111^IXXXI^115^ the ability to accumulate Cu and activate a Cu-responsive promoter was impaired [43]. No data are available on the importance of the methionines in the extracellular N-terminus of CTR2 although inference from yeast and the conserved nature of these residues would also suggest these are essential for Cu transport as well. There are other residues within CTR2 that are conserved across species that also differ from CTR1, such as the C-terminal double leucine, but the functional significance of these has not been investigated to date.

While CTR1 resides entirely outside the nucleus, CTR2 is found both in the endosomal/lysosomal compartment and also within the nucleus in a punctuate distribution not immediately recognizable as being similar to that of any other nuclear protein as determined by immunocytochemical staining and deconvoluting microscopy [45]. This finding has been confirmed by western blot analysis of isolated nuclei. It is tempting to speculate that nuclear CTR2 is involved in the regulation of nuclear Cu levels although it is unlikely that nuclear CTR2 is associated with membranes given its location and distribution within the nucleus, and it thus seems doubtful that it is functioning as a metal transporter at this site. CTR2 does not contain any obvious nuclear localization signal or DNA binding motifs, and it likely interacts with other proteins to reach the nucleus. Its presence in the nucleus also begs the question of how a transmembrane protein could be present in a soluble form. However, the epidermal growth factor receptor (EGFR) and CD44 provide precedent for the finding of transmembrane proteins in the nucleus [46, 47].

## 6. Regulation of Cu and Pt Drug Cytotoxicity by CTR2

Initial experiments on mammalian CTR2 showed that it was capable of importing Cu into the cell under conditions of excess extracellular Cu; little effect was observed at low Cu concentration (~2 *μ*M) [8, 43]. These experiments used systems in which CTR2 was forcibly over-expressed and the ability of CTR2 to mediate Cu import has not been consistently reported. More recent work showed that when CTR2 was stably knocked down there was a modest increase in Cu uptake but little effect on Cu toxicity [25]. This effect was even less apparent in CTR1^−/−^ cells, and CTR2 knockdown was associated with little effect on Cu cytotoxicity regardless of CTR1 expression further suggesting that CTR2 plays little role in the import of extracellular Cu.

Given the structural similarities between CTR1 and CTR2, studies were undertaken to determine whether CTR2 played any role in transport of the Pt-containing drugs. Experiments performed in mouse embryonic fibroblasts showed that knockdown of CTR2 had a large effect on the accumulation of both cisplatin and carboplatin [25]. However, instead of limiting import, knockdown of CTR2 increased the uptake of both cisplatin and carboplatin by a factor of 2-3-fold, an effect opposite to that of knocking out CTR1. This increase in Pt drug uptake translated to an increase in formation of Pt-DNA adducts and increased cytotoxicity. The increased uptake was found to be due to enhanced initial influx rather than an effect on efflux and was independent of CTR1 as a similar increase occurred when CTR2 was knocked down in CTR1^−/−^ cells. No change was detected in the concentration of Pt in the microvesicular fraction, although as whole cell Pt increased a smaller fraction of total intracellular Pt was found in this fraction suggesting impaired loading of Pt into this compartment. Thus, it appears that CTR1 and CTR2 have opposite effects on Pt drug uptake. As of yet, the mechanism by which CTR2 controls Pt drug uptake is still unclear. Whether the effect of CTR2 involves direct transport or is secondary to effects on other import pathways is unknown. However, the fact that the knockdown of CTR2 causes a similar increase in cisplatin uptake in the presence or absence of CTR1 suggests that it is involved in a non-CTR1 dependent mechanism of uptake.

## 7. Regulation of CTR2 Expression by Cu and Cisplatin

The level of CTR1 is altered by exposure to Cu or cisplatin, and this appears to be true for CTR2 as well. Exposure of many types of cells to high concentrations of Cu, or low concentrations of cisplatin, results in the downregulation of CTR1 by a process that involves macropinocytosis, ubiquitination, and proteasomal degradation [48–50]. This has been interpreted as a mechanism that helps limit the toxicity of high extracellular levels of Cu. CTR2 expression is also affected by exposure to Cu and cisplatin but there are discrepancies between various systems in which this has been examined. In the green alga *Chlamydomonas reinhardtii* and the fungus *Colletotrichum gloeosporioides* CTR2 expression was found to decrease in the presence of high extracellular levels of Cu [51, 52]. In contrast, in mammalian cells exposure to either Cu or cisplatin resulted in an increase in CTR2 mRNA and protein levels [45]. The difference between the effect of Cu in mammalian cells versus that in algae and fungus may reflect species differences and variation in nomenclature. It is notable that CTR2 in *Colletotrichum* is actually more closely related to human CTR1 when examined by BLAST sequence alignment consistent with this contention and even contains additional N-terminal extracellular metal binding sequences similar to hCTR1 that are lacking in hCTR2. The protein named CTR2 in *Chlamydomonas*, while also mediating Cu transport, is several times larger than CTR2 in mammalian cells being over 800 amino acids in length. It also contains different types of metal binding sequences and appears to have been named “CTR2” based more on the fact that it was the second CTR family protein described in that species [51] rather than on the basis of its similarity to CTR2 in other species.

The regulation of CTR2 expression occurs at both transcriptional and posttranscriptional levels as mRNA levels as well as protein half-life are affected. Exposure to 30 *μ*M cisplatin for one hour increased both the mRNA level and the protein level by ~1.5-fold whereas treatment with 100 *μ*M Cu increased both mRNA and protein levels by ~3-4-fold at 1 hour [45]. This increase in protein level was accompanied by an increase in protein half-life by 3.1-fold to 43.6 minutes in cisplatin-treated cells and by 1.6-fold to 22.7 minutes in Cu-pretreated cells. This posttranscriptional regulation is dependent on the Cu chaperone ATOX1 as ATOX1^−/−^ cells are unable to up- or down-regulate CTR2 following exposure to Cu, cisplatin, or the Cu chelating agent bathocuproine disulphonate (BCS). Although ATOX1 has been shown to function in the nucleus to regulate transcription [11] and there are some putative ATOX1 binding sites in the CTR2 promoter, the regulation of CTR2 by ATOX1 appears to be at the posttranscriptional level as mRNA levels still increase or decrease in response to drug exposure in a manner similar to that in ATOX1^+/+^ cells [45]. The decreased CTR2 protein half-life observed following Cu starvation is at least in part due to increased proteasomal degradation as the proteasome inhibitor bortezomib can block this effect; interestingly, bortezomib also blocks the degradation of CTR1 induced by cisplatin exposure [50]. The details of the mechanism by which ATOX1 controls the Cu- or cisplatin-induced regulation of either CTR1 or CTR2 remain to be worked out.

## 8. Discussion

Many of the proteins involved in Cu homeostasis influence the uptake and cytotoxicity of the Pt-containing drugs [1, 21, 53], and cisplatin has been shown to interact with several of these including the efflux pump ATP7B [54]. The current model of Pt drug uptake suggests that Cu transporters and chaperones mediate drug uptake and efflux in a manner similar to Cu [30]. The observation that reduction in the expression of CTR1 and CTR2 has opposite effects on the cytotoxicity of Cu and cisplatin, and that both Cu and cisplatin regulate CTR1 and CTR2 in opposite directions, suggests that these two transporters function quite differently in the cell and do not simply participate in redundant pathways of Cu influx. Whereas CTR1 mediates accumulation of the Pt-containing drugs, CTR2 appears to limit their influx in mouse embryo fibroblasts. 

The evidence that CTR1 controls uptake of the Pt drugs is now quite strong and has been confirmed by multiple laboratories [24, 31, 55–58]. However, the evidence that CTR2 regulates Pt drug accumulation has come from just one laboratory and requires verification. Although a model of how the Pt drugs might pass through the pore formed by CTR1 via a series of transchelation reactions has been proposed [59], there remains the possibility that CTR1 delivers cisplatin into the cell also by chelating it via the N-terminal methionines followed by endocytosis or that CTR1 regulates a separate Pt drug influx mechanism. This different mechanism could be dependent on Cu, and the observed decrease in cisplatin uptake in CTR1 mutants could be due to a derangement in Cu metabolism. Not unexpectedly, given the differences in their structure, there is now evidence for substantial differences in the details of how Cu and cisplatin interact with CTR1 [34, 40, 60]. 

Based on its structural similarity to CTR1, its ability to modulate Cu influx when situated in the plasma membrane, and its ability to export Cu from the yeast vacuole, it is very likely that CTR2 also forms a pore in cellular membranes through which Cu passes. CTR2 limits cisplatin uptake and this appears to be the result of reduced initial influx rather than enhanced efflux. This effect of CTR2 is independent of CTR1 as knockdown of CTR2 produced the same effect in both CTR1^+/+^ and CTR1^−/−^ cells. This argues against the idea that CTR1 and CTR2 act in series to mediate cisplatin accumulation. Based on its role in *S. cerevisiae *one might imagine that the primary function of CTR2 is to shuttle Cu and the Pt drugs between intracellular compartments. However, the finding that Pt accumulation in the microsomal fraction remained unchanged in cells in which the expression of CTR2 was knocked down argues against a role for CTR2 in either sequestering or effluxing cisplatin from vesicles as this would have been expected to alter the microsomal content of Pt which was not observed [25]. Thus, how CTR1 enhances and CTR2 limits Pt drug uptake remains enigmatic at present. 

Given that CTR2 has such a large effect on Pt drug uptake and cytotoxicity, the pharmaceutical regulation of CTR2 expression may allow the efficacy of the Pt drugs to be manipulated to enhance selectivity. One potential way in which this might be achieved is through the use of Cu chelators to decrease the level of CTR2 expression and thus increase Pt drug uptake. Indeed, this very method has recently been shown to improve cisplatin efficacy in an *in vivo* mouse cervical cancer model in which pretreatment with the Cu chelator tetrathiomolybdate was shown to increase the uptake of cisplatin and tumor killing [61]. 

Regardless of the mechanisms by which CTR1 and CTR2 transport the Pt drugs, there is little doubt that they have relatively large effects on the cytotoxicity of cisplatin both *in vitro* and *in vivo*. A potential clinical application has already emerged from studies of how the Cu homeostasis proteins influence the cellular pharmacology of the Pt drugs. A clinical trial of intraperitoneal pretreatment with bortezomib prior to the intraperitoneal administration of carboplatin in patients with ovarian cancer, directed at preventing carboplatin-induced CTR1 downregulation, has just been opened. A more detailed understanding of the mechanism by which Cu homeostasis transporters and chaperones influence the cytotoxicity of the Pt drugs has a high probability of resulting in further improvement of the therapeutic index of this important class of chemotherapeutics.

## Abbreviations

BCS: Bathocuproine disulphonate
CTR1: Cu transporter 1, SLC31A1
CTR2: Cu transporter 2, SLC31A2
cDDP: Cisplatin.
